# Effect of a family-based lifestyle intervention on weight reduction among Jordanian children with obesity aged 6–9 years

**DOI:** 10.29219/fnr.v68.9582

**Published:** 2024-05-13

**Authors:** Ayman K. Bani Salameh, Mamdouh El-Hneiti, Omar S.H. Al Omari, Mohamed AlBashtawy, Savvato Karavasileiadou, Yasmine Alabbasi, Khlood Saleh Bubshait, Malakeh Z. Malak

**Affiliations:** 1 Pediatric Health Nursing, Al-Zaytoonah University of Jordan, Amman, Jordan; 2 Community Health Nursing, Faculty of Nursing, Jordan University, Amman, Jordan; 3 Pediatric Health Nursing, College of Nursing, Sultan Qaboos University, Muscat, Oman; 4 Community Health Nursing, Princess Salma Faculty of Nursing, Al al-bayt University, Almafraq, Jordan; 5 Community, Psychiatric, Mental Health Nursing Department, College of Nursing, Princess Nourah Bint Abdul Rahman University, P.O Box 84428, Riyadh 11671, Riyadh, Saudi Arabia; 6 Department of Maternity and Child Health Nursing, Princess Nourah Bint Abdulrahman University, P.O Box 84428, Riyadh 11671 Riyadh, Saudi Arabia; 7 Fundamental of Nursing Department, Imam Abdulrahman Bin Faisal University, Dammam, Saudi Arabia; 8 Community Health Nursing, Faculty of Nursing, Al-Zaytoonah University of Jordan, Amman, Jordan

**Keywords:** children, dietary habits, family-based intervention, obesity, physical activity

## Abstract

This study aimed to evaluate the effect of a family-based lifestyle intervention on reducing body weight among Jordanian children with obesity aged 6–9 years old. The pretest-posttest control group design was conducted among 162 children (75 in the intervention group and 87 in the control group) with obesity aged 6–9 years old at four primary schools in Jordan during the period from March 2021 to July 2021. The results found that, after the intervention, there was a statistically significant change in the *F* scores in the control group vs. in the intervention group (*M* = 37.07, SD = 2.77; *M* = 33.48, SD = 2.73; *t* (160) = 8.29, *P* < 0.001), where the mean BMI percentile was reduced by 2.05 in the intervention group. A significant difference was demonstrated in the median BMI percentile in the intervention and control groups post-intervention (*P* < 0.001). A significant difference was also noticed between the average weekly reported dietary habits and the physical activities of both the control group and the intervention group post-intervention. The findings support the effect of family-based lifestyle interventions. Healthcare providers should adopt such interventions for children living with obesity. Future study is required to evaluate the long-term effectiveness of this intervention on weight reduction.

## Popular scientific summary

- Obesity and overweight among children is worldwide increasing, specifically in aged 6-9 years. This study found that a family-based lifestyle interventions can reduce the BMI percentile, enhance healthy dietary habits and increase physical activity. Such interventions need to be adopted for children with obesity in shcools.

Childhood obesity is a worldwide health issue that is acknowledged by the World Health Organization (1). Overweight and obesity among children are alarming health issues, particularly, in both developing and developed countries (2), because they have become one of the leading causes of morbidity and premature mortality (3). In 2016, the World Health Organization (1) estimated that, globally, 18% (approximately 340 million) of children and adolescents aged 5 to 19 years old were overweight or obese. In Jordan, overweight (17.3%) and obesity (15.7%) among school age children are considerably high as it is reported by the World Health Organization (4).

During the school age (from ages 5 to 8), females need around 1,200 to 1,800 calories per day, and males need around 1,200 to 2,000 calories per day. School-aged children and adolescents require at least 60 min of moderate to intensive exercise per day to have the most health values from exercise. Most exercises should focus on aerobic, like walking, running, or any activity that increases heartbeat. School-aged children and adolescents also require to do activities, which strengthen their bones and muscles, such as play with climbers, basketball, and skipping rope (5). However, according to the WHO (4), female has reported more overweight than male (18.9 and 15.3%, respectively), while male reported more obesity than female (18.9 vs. 11.2%).

Children’s psychosocial health is influenced by overweight and obesity. Children with obesity and overweight may suffer from low self-self-confidence and sadness during adolescence. In addition, bad psychological experiences, for instance, prompt stress eating, which cause an increased calorie intake, with a consequent increase in the risk of obesity (6). Children who are overweight or obese may be exposed to bullying at school and might be excluded from competitive physical activities. Overall, children with obesity may experience less social interaction and may spend more time in sedentary activities. One study confirmed the relationship of childhood obesity with attention deficit hyperactivity disorder and anxiety disorders (6).

Obesity is caused by many factors, including increased consumption of energy-dense foods; excessive sugar and soft drinks; lack of physical activity; socioeconomic factors related to families, such as low parental education, urbanization, and modernization (7); as well as from sedentary lifestyles (7, 8), for example, spending too much time watching television and playing video games (9, 10). Other risk factors are genetic, such as a family history of parenteral obesity (9) and the attitudes of children and parents toward obesity and overweight (11, 12).

Childhood obesity is defined as a serious health problem that impacts on school age children. It is a big issue as the additional weight usually begins children on the way to health issues which were once thought adult health problems, such as diabetes mellitus, hypertension, and high cholesterol (13). Childhood obesity leads to many physical problems, including cardiac problems, type 2 diabetes mellitus (14), metabolic syndrome, and neurological, pulmonary, orthopedic, hepatic, and renal diseases (15). It is also responsible for some psychological consequences, such as low self-esteem, poor social health and emotional well-being, and low quality of life (16). Furthermore, childhood obesity is associated with low educational achievements (17) and can lead to adult obesity (18).

Given the high incidence of obesity among children and the response to the negative effects of childhood obesity, previous research has explored many effective and successful interventions to reduce the weight of children aged 2–15 years. For example, earlier studies evaluated the impact of adopting healthy dietary habits, such as enhanced consumption of fruits and vegetables (19, 20) and increased physical activity (21, 22). Other studies have focused on enhancing external factors, such as the availability of the environment and necessary facilities to promote physical activities, including the availability of gyms, landscapes, sidewalks, and/or footways (23–25). However, some approaches to intervention have met with limited success in reducing BMI percentile scores, while they have enhanced dietary habits and physical activity (25).

The ultimate goal of the previous interventions was to help children lose weight. Several interventions were developed to meet this goal. One of these interventions is the family-based lifestyle intervention, which was developed to prevent the development of childhood obesity and to help children reduce weight (26) by encouraging physical activity and balanced diets (27). However, because of the high prevalence of obesity among children, this initiative needs evaluation and further development to target overweight and obese children. Another famous method of weight reduction among children is the family-based lifestyle intervention that includes behavioral methods, which has been indicated as an initial intervention and is vital for long-term weight reduction and control (28, 29).

Several polls have accepted the effectiveness of family-based lifestyle interventions involving behavioral methods (30, 31). However, up to our knowledge, there is a lack of studies about the effectiveness of family-based lifestyle interventions on children with obesity aged 6 to 9 years old in the Eastern Mediterranean region, which includes Jordan. There is a single study that examined the effect of family-based interventions on Jordanian adolescents with obesity and proved its effectiveness in reducing weight (31).

This region, including Jordan, has different cultural, eating, and family/lifestyle habits from other countries in which researchers have studied children’s weight reduction interventions. Many Jordanians prefer to eat meat and fats and do not exercise regularly, and some families do not care about the types or quantities of food consumed by their children (31). In Jordan, children are more likely to be less active and tend to spend more time playing video games, TV, and computers. However, those children who are living in a village are more likely to be more active and tend to play more with their friends in the neighborhood (32).

Therefore, this study could help healthcare providers, policymakers, the community, and families to adopt this intervention that requires family collaboration and lifestyle modifications to reduce weight through promoting healthy dietary habits and physical activity.

## Objectives and hypothesis

The primary objective of the current study was to evaluate the effect of a family-based lifestyle intervention on reducing body weight among Jordanian children with obesity aged 6 to 9 years old. The secondary objective was to evaluate the change in lifestyle outcomes (dietary habits and physical activity) in Jordanian children with obesity aged 6 to 9 years old after the implementation of the intervention.

There were two hypotheses tested in this study. First, the Jordanian children with obesity and their parents who engaged in a family-based lifestyle intervention would demonstrate greater reductions in weight compared to those who did not engage in a family-based lifestyle intervention. Second, the Jordanian children with obesity and their parents who engaged in a family-based lifestyle intervention would demonstrate greater changes in lifestyle outcomes (dietary habits and physical activities) compared to those who did not engage in a family-based lifestyle intervention.

## Methodology

### Design

The pretest-posttest control group design was conducted in Amman, the capital of Jordan, at four governmental schools. Clustering was chosen because it is the feasible way to implement the study intervention at the school level. A cluster random sampling method was carried out, in which the Amman governorate was divided into four zones: north, east, south, and west. Then, the researchers selected only governmental primary schools, which referred to the Ministry of Education. Then, one school was selected from each zone from a list given by the managers of the educational departments in Amman using a simple random method. After that, two schools were randomly chosen to be in the intervention group, and the other two schools were chosen to be in the control group.

## Tool/instrument

### Population, sample, and sample technique

The students in the same two schools were allocated to the intervention group, which received the family-based lifestyle intervention, and the students in the other two schools were allocated to the control group, which did not receive any treatment. All students were followed for 3 months and received two outcome measurements. Evaluations were performed at the baseline and 3 months later.

### Participants and randomization

The study was conducted in the schools located in Amman, the capital of Jordan. The governmental schools were chosen to be representative because of their socioeconomic status. The study included 162 students with obesity, aged 6 to 9 years old who were interested in family-based lifestyle interventions and was conducted from March 2021 to July 2021. The students who met the following inclusion criteria were enrolled: 1) aged between 6 and 9 years old, 2) obese (with BMI in the 95^th^ percentile or more), and 3) willing to participate in this study.

## Inclusion and exclusion criteria

Exclusion criteria included children who 1) were unable to participate in the organized interventional activity, 2) had undertaken any obesity management program, or 3) had been diagnosed with a developmental or mental disorder, autism or psychosis, genetic obesity syndrome, or any other contraindication.

### Sample size

The G* Power (3.0.10) program was adopted to calculate the sample size. The power analysis indicated that a minimum sample of 73 students from each group would be enough for the *t*-test to detect a medium effect size (0.50), with the α level of 0.05 and power of 0.85. To avoid dropouts, there was a 25% increase in enrollments, to reach at least 184 participants, who were then divided into two groups.

The researchers revised the health records of students sequentially in the respective schools. The records of the participants who met the inclusion criteria were retrieved and included in one file. The eligible students and caregivers (guardians and parents), who were agreed to participate, were provided with consent forms, and the children gave assent. After assuring the homogeneity of the subjects using statistical tests, the researchers randomly pulled out the names of potential participants who met the inclusion criteria to be either in the intervention group or in the control group. The file names were kept concealed in closed envelopes and in private files on the computer.

### Outcome measures

The main outcome of this study was related to weight reduction. Also, the secondary lifestyle outcomes involved dietary habits and physical activity. The weight reduction was assessed using the BMI-for-age percentile and median BMI percentile. The BMI was measured by dividing the body weight by the height in meters squared. Finally, we calculated the BMI percentile, which is considered a measure of children’s weight according to their age and sex. The BMI was classified as follows: underweight (BMI falls under the 5^th^ percentile), normal weight (BMI falls in the 5th–85th percentile), overweight (BMI falls into or above the 85^th^ percentile but below the 95^th^ percentile), and obese (BMI falls within or over the 95^th^ percentile) (33). The obesity percentage was also calculated using the median BMI for gender and age (34). The BMI *Z*-score was not used because of its weakness as a predictor of changes in body fat adiposity (35).

Lifestyle outcomes, including dietary habits and physical activity, were assessed by a questionnaire consisting of questions related to dietary habits (seven questions including four questions about unhealthy dietary habits and three questions about healthy dietary habits) and physical activity (eight questions involving five questions about active exercises and three questions about inactive exercises). This questionnaire was developed by Bani Salameh (36) on adolescents (31). The Arabic version of this tool is valid and reliable, with an internal reliability of Cronbach’s alpha > 0.70 (31), and it was modified to be appropriate for children in this study by a reduction in the number of original questions for dietary habits from 15 to seven and for physical activity from 15 to eight in the new version to be collected daily, and the averages were calculated for 1 week.

A pilot study was conducted on children with obesity aged 6 to 9 years old (*N* = 15), which showed that all items in the questionnaire were clear and understandable. It took approximately 10 min for the children to complete the questionnaire. The internal consistency for reliability was obtained, in which Cronbach’s alpha was 0.78.

### Family-based lifestyle intervention

The intervention was lifestyle education, which aimed at encouraging children to enhance healthy dietary habits and to increase physical activity in order to reduce their body weight. The intervention was adopted from a ‘Swap It, Don’t Stop It’ (SIDSI) program (originally developed by the Australian government), which was modified and adapted taking into account the culture (36) and had been piloted on Jordanian adolescents in 2012–2013. Participants were delivered the modified Arabic version.

The intervention was delivered over 3 months (12 weeks), every 2 weeks in the auditoriums of the schools. The principals of the schools helped with the research by providing resources, such as a hall, a data show, and a whiteboard. In addition, the homeroom teachers facilitated the selection of the participants and arranged for the meetings with the parents.

A letter of invitation to parents was sent out with the participants 3 days before each session in which their cooperation and understanding were important. They attended the intervention sessions every 2 weeks at the schools after classes were over. The rationale for children attending was to encourage them to adopt healthy dietary habits and to increase physical activity through behavioral modifications.

The intervention consisted of six sessions (30 min each with 10–11 participants in each one as well as their parents), which were followed by phone calls for reassurance if needed. These sessions were led by three of the researchers. The interventions consisted of educational sessions about dietary habits, physical activity, and behavioral methods. The participants were taught about such things as dietary habits, including food quality and quantity, and encouragement to practice physical activity for around 7 h per week and to decrease screen games and watching television to less than 14 h per week. Additional instruction was given about behavioral methods directed toward children and included motivations, such as promoting positive body image, providing positive reinforcement, and rewarding achievement of the planned goals (31, 36). The intervention sessions and their components are presented in Table 1.

**Table 1:** Intervention sessions and components

Number of Session	Components	Contents	Educator
1	Lifestyle outcomes (Dietary habits & Physical activity)	Obesity: Causes and consequences Food quality and quantity (portion size and the types of food). Importance of physical activity.	Researchers
2	Lifestyle outcomes (Dietary habits & Physical activity) Behavioural methods	Food pyramid Classifications and types of exercises (vigorous, moderate, and low). Motivation and reinforcement	Researchers
3	Lifestyle outcomes (Dietary habits & Physical activity) Behavioural methods	Reassurance using SIDSI food guidelines (portion size and food type) Reassurance using SIDSI physical activity guidelines	Researchers
4	Lifestyle outcomes (Dietary habits & Physical activity) Behavioural methods	Bad eating habits (sweet snacks, fizzy drinks, and junk food) Minimizing inactive habits (study-at-home screen time, recreation screen time, and sleeping/ socializing/ relaxing during daytime) Motivation and reinforcement	Researchers
5	Lifestyle outcomes (Dietary habits & Physical activity) Behavioural methods	Food nutritional values (healthy food) Calories burned each hour from different activities. Motivation and reinforcement	Researchers
6	Lifestyle outcomes (Dietary habits & Physical activity) Behavioural methods	Meal skipping Importance of maintaining regular physical activity Motivation, reinforcement, and rewards	Researchers

At the end of each session, the participants and their parents in each group were offered complimentary education materials, such as a manual of daily diet and physical activity suggestions. Participants were instructed to bring their own healthy breakfast and snacks from home. These meals would then be prepared under parental supervision. Participants were also encouraged not to buy any junk food sold at the school market or from markets around the school.

### Data collection

After we obtained ethical approval, the children living with obesity were selected, with assistance from the homeroom teachers, according to their observed body types (endomorphic). A baseline Center for Disease Control and Prevention (CDC) BMI-for-age percentile and median BMI percentile were measured for both groups (intervention and control) before the intervention. Children were weighed fortnightly by three of the research team in the morning before breakfast, without shoes and with light clothing on a digital scale (Garmin Index Smart Scale). A fixed height board was used for height measurements. The height and weight were measured by placing each child in the middle of the scale with minimal clothing to measure their weight (4).

The total number of selected students was 363, of which 179 students did not meet the inclusion criteria and not being willing to participate. The qualified participants for the intervention (*n* = 184) were met by one of the research team to explain the research purposes and to determine their interest in participating in the study through arranged meetings in the school auditoriums. If the children and parents were interested, the parents completed an informed consent form and entered demographic data.

The questionnaires were administered separately to the control group (children and their parents) and to the intervention group but at the same time point before the first session. After the parents and children completed the questionnaires before the meeting, the researchers collected them.

This questionnaire included assessments for dietary habits 1 week before the intervention by calculating their 24-h recall of meals for the average of these recalls for 1 week. Physical activities were also assessed by calculating the 1-week average of the demonstrated activities for 1 week before the intervention. Then, the intervention sessions were conducted for the intervention group.

In the beginning, the intervention group was divided into eight groups, and, for cultural considerations, these groups were divided according to gender (four groups of females and four groups of males). The intervention group attended the family-based lifestyle intervention for 3 months, completing the questionnaire 1 week after the intervention, and the BMI-for-age percentile and median BMI percentile were then recalculated. The control group did not attend any interventions, but they were asked to complete the questionnaires after the intervention group completed the intervention, and their BMI-for-age percentile and median BMI percentile (post-intervention) were also remeasured.

Data were collected from March 2021 to July 2021. The data were collected before the intervention and 3 months after the intervention (post-intervention) in a paper form at the school auditoriums. Three members of the research team were trained by the main researcher and took responsibility for data collection.

For ethical considerations, care was provided to the control group, in this the group was exposed to the intervention by administering three extensive sessions (60 min for each session) after the completion of the study.

### Ethical considerations

The approval to conduct this study was obtained on 10 December 2020 from the Institutional Review Board at Al-Zaytoonah University of Jordan with reference number (03/145/2020–2021). Then, ethical approval was obtained from the Ministry of Education. All procedures performed in studies involving human participants were in accordance with the ethical standards of the institutional and/or national research committee and with the 1964 Declaration of Helsinki and its later amendments for comparable ethical standards. The information sheet, which detailed the purpose, benefits, and risks of the study, and a demographic assessment sheet were sent home along with the consent forms with each child to the parents/guardians as a primary study invitation to participate. Confidentiality was assured in all stages of the research. All subjects were informed that they could withdraw from the study at any time if they wished not to participate, and they would have no harmful effects.

### Statistical analysis

The SPSS version 23 software program was used for data entry and analysis of the data. We used the SPSS Missing Value Analysis package to estimate the pattern of missing data and to input missing values by the appropriate procedures. There were missing values in the data for neither the control group nor the intervention group.

Levene’s test of equality of error variances and Chi-square and independent *t-*tests were used to test for differences and for the homogeneity of the sample, respectively. Levene’s test of equality of error variances showed that all the demographic variables had equal variances (*P* < 0.05). Furthermore, Chi-square tests showed that there were no differences between the groups in relation to gender *(X_2 (1, 162)_ = 1.45, P = 0.22)*, educational level of fathers *(X_2 (5, 162)_ = 9.43, P = 0.093)*, educational level of mothers (*X_2 (5, 162)_ = 6.422, P = 0.267)*, or income *(X_2 (5, 162)_ = 0.65, P = 0.74)*. Also, independent *t*-tests showed that there were no differences in the mean age between the groups (*M = 7.45 (SD = 1.06); t _(160)_ = –0.326, P = 0.745*) and BMI percentile scores (Table 2).

**Table 2:** Demographic characteristics of study participants (*N* = 162)

Characteristics	Intervention Group *n* = 75 (46.3%)	Control Group *n* = 87 (53.7%)	Total	Statistical test	
	M(SD)	M (SD	M(SD)	*t*-test	*p*-value
Age	7.40 1.09	7.48 (1.03)	7.45 (1.06)	–0.326	0.745
	*n* (%)	*n* (%)	*n* (%)		
**Gender**				**Chi-square**	***p*-value**
Male	37 (49.3)	44 (50.6)	40.5 (50.0)	1.45	0.22
Female	38 (50.7)	43 (49.4)	40.5(50.0)		
**Fathers’ educational level**				**9.43**	**0.093**
Primary	4 (5.3)	3 (3.4)	3.5(4.3)		
Secondary	16 (21.3)	11 (12.6)	13.5 (16.7)		
Diploma	16 (21.3)	11 (12.6)	13.5 (16.7)		
BSc	20 (26.7)	38 (43.7)	29 (35.8)		
Higher education	19 (25.3)	24 (27.5)	21.5 (26.5)		
**Mothers’ educational level**				6.422	0.267
Primary	10 (13.3)	5 (5.7)	7.5 (9.3)		
Secondary	28 (37.3)	29 (33.3)	28.5 (35.2)		
Diploma	10 (13.3)	20 (23)	15.0 (18.5)		
BSc	19 (25.3)	23 (26.4)	21.0 (25.9)		
Higher education	8 (10.6)	10 (11.5)	9.0 (11.1)		
**Socioeconomic status**				0.65	0.74
Upper middle class	14 (18.7)	14 (16.1)	14 (17.4)		
Middle class	42 (56.0)	50 (57.5)	67 (56.8)		
Lower middle class	19 (25.3)	23 (26.4)	21 (25.8)		

*n*: number; %: percentage.

M: Mean; SD: Standard Deviation.

*p*-value: *significant at < 0.05 level; **Significant at < 0.001 level.

Furthermore, descriptive statistics were used to analyze the demographic data, where mean and standard deviation were used for continuous variables and number and percentage (%) for categorical variables. Independent *t*-tests were used to test for differences between the intervention and control groups in BMI percentile, and the Wilcoxon signed rank test and Mann-Whitney test were used to test the differences between the median BMI percentiles. The level of significance of ≤ 0.05 was used.

## Results

### Participants’ flow

**Fig. 1:**
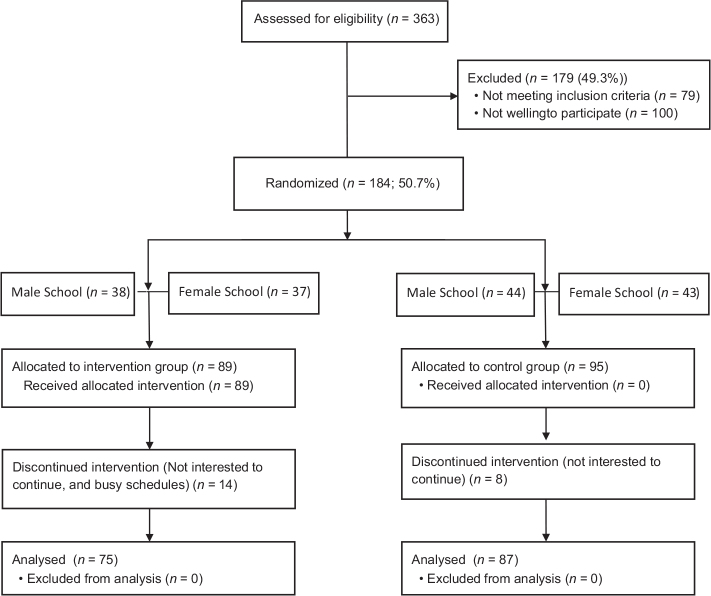
Participants’ flow in this study.

Figure 1 illustrates the participants’ flow in this study. In the beginning, 184 eligible students with their parents were approached for recruitment into the study, and 184 students consented to participate. However, the final sample was 162 participants who took part in the current study because 23 participants (8 participants (8.4%) from the control group and 14 participants (15.7%) from the intervention group) dropped out after the first measurement. The reasons for dropout were the same for both groups, such as the participants showing no interest in continuing the study or busy schedules. One hundred sixty-two participants (87 control and 75 intervention) completed the study.

### Participants characteristics

Regarding the participants’ demographic characteristics, the mean age of the participants was 7.45 years (SD = 1.63), including 53.1% (*n* = 86) females and 46.9% males (*n* = 76). For more details, see Table 2.

### Intervention effect on BMI percentile, median BMI percentile, dietary habits, and physical activity

Table 3 illustrates that there were no significant differences in the mean BMI percentile scores between the groups before the intervention (*M = 35.61, SD = 3.03; M = 35.53, SD = 2.39; t _(160)_ = 0.18, P = 0.858*, respectively) with a 95% confidence interval (CI). However, there was a statistically significant positive change in mean BMI percentile scores before and after intervention (*M = 35.53, SD = 2.39; M = 33.48, SD = 2.73; t _(160)_ = 16.78, P* < 0.001) with a 95% CI for the participants in the intervention group, where the mean BMI percentile score was reduced by 2.05. However, there was a significant negative change in the mean BMI percentile before and after implementing the intervention (BMI percentile > 99%) for the control group (*M = 35.61, SD = 3.03; M = 37.07, SD = 2.77; t (160) = –12.84, P < 0.001*) with a 95% CI, in which the children in the control group had an increase in BMI percentile. Additionally, there was a statistically significant difference in the mean BMI percentile scores between the control and intervention groups after the intervention (*M = 37.07, SD = 2.77; M = 33.48, SD = 2.73; t _(160)_ = 8.29, P < 0.001*, respectively) with a 95% CI. Furthermore, there was a significant difference between the intervention and control groups post-intervention in the median BMI percentile, in which the weight reduction in the intervention group was statistically significantly higher than that in the control group (*Mann-Whitney U = 1235.50, P < 0.001*) with a 95% CI.

**Table 3:** Change in BMI percentile scores and median BMI percentile in intervention and control groups across pre and post-intervention

	Control group (*n* = 87)			Intervention group (*n* = 75)			Difference between groups	
	Pre	Post	*t*-test *p*-value	Pre	Post	*t*-test *p*-value	Pre	Post
	M (SD)	M (SD)		M (SD)	M (SD)		*t*-test *p*-value	*t*-test *p*-value
BMI percentile	35.61 (3.03)	37.07 (2.77)	*t* = –12.84 *p* < 0.001**	*t* = 35.53 (2.39)	33.48 (2.73)	*t* = 16.78 *p* < 0.001**	*t* = 0.18 *p* = 0.858	*t* = 8.29 *p* < 0.001**
	**Median**	**Median**	**Z^a^** ***p*-value**	**Median**	**Median**	**Z^a^** ***p*-value**	**U** ***p*-value**	**U** ***p*-value**
Median BMI percentile	34.60	35.80	–3.571b *p* < 0.001**	35.34	32.82	–7.324c *p* < 0.001**	U = 3052.00 *p* = 0.479	U = 1235.50 *p* < 0.001**

M: Mean; SD: Standard Deviation.

BMI percentile: Body Mass Index percentile.

Pre: pre-intervention. Post: post-intervention.

*P*-value:*significant at < 0.05 level; ** Significant at < 0.001 level.

a Wilcoxon Signed Ranks Test.

b. Based on negative ranks.

c. Based on positive ranks.

U: Mann-Whitney U.

Tables 4 and 5 represent the children’s average weekly reported dietary habits and their reported physical activity before and after implementing the intervention. There were no significant differences between the children in the control and intervention groups in the pre-intervention stage. However, a significant difference between the groups in dietary habits and physical activity was noticed after the intervention.

**Table 4:** Average weekly dietary habits across groups in pre and post- intervention

Dietary habits change								
Item	Control group (*n* = 87)			Intervention group (*n* = 75)			Difference between groups	
	Pre	Post	*t*-test *p*-value	Pre	Post	*t*-test *p*-value	Pre	Post
	M SD	M SD		M SD	M SD		*t*-test *p*-value	*t*-test *p*-value
No. of meals.	4.86 (1.03)	4.93 (1.53)	*t* = –0.38 *p* = 0.709	5.27 (1.25)	3.45 (0.64)	*t* = 14.79 *p* < 0.001**	*t* = –2.26 *p* = 0.025*	*t* = 7.79 *p* < 0.001**
No. of large meals.	3.71 (0.81)	4.33 (0.82)	*t* = –5.84 *p* < 0.001**	3.71 (0.90)	1.33 (0.47)	*t* = 20.90 *p* < 0.001**	*t* = 0.05 *p* = 0.964	*t* = 27.99 *p* < 0.001**
No. of pieces of fruit.	2.05 (1.20)	2.02 (1.07)	*t* = 0.146 *p* = 0.884	2.25 (0.97)	4.32 (0.93)	*t* = –19.45 *p* < 0.001**	*t* = –1.19 *p* = 0.234	*t* = –14.47 *p* < 0.001**
No. of serves of vegetables.	1.48 (0.70)	1.38 (0.61)	*t* = 1.09 *p* = 0.281	1.84 (1.0)	3.64 (0.80)	*t* = –20.22 *p* < 0.001**	*t* = –2.66 *p* = 0.008**	*t* = –20.32 *p* < 0.001**
No. of glasses of water.	3.71 (0.81)	3.25 (0.84)	*t* = 4.64 *p* < 0.001**	3.71 (0.90)	6.05 (1.11)	*t* = –23.89 *p* < 0.001**	*t* = 0.05 *p* = 0.006**	*t* = –18.22 *p* < 0.001**
No. of snacks of sweet/ chocolates.	3.51 (0.75)	4.24 (0.79)	*t* = –8.43 *p* < 0.001**	3.71 (0.90)	1.24 (0.43)	*t* = 21.27 *p* < 0.001**	*t* = –1.55 *p* = 0.121	*t* = 29.30 *p* < 0.001**
No. of glasses of sweet fizzy drinks.	3.30 (0.99)	3.98 (0.84)	*t* = –7.79 *p* < 0.001**	3.48 (0.94)	1.24 (0.43)	*t* = 18.93 *p* < 0.001**	*t* = –1.19 *p* = 0.235	*t* = 25.53 *p* < 0.001**

M: Mean; SD: Standard Deviation.

Pre: pre-intervention. Post: post-intervention.

*p*-value: *significant at < 0.05 level; ** Significant at < 0.001 level.

**Table 5:** Average weekly physical activity across groups in pre and post- intervention

Item	Means of physical activity change							
	Control group (*n* = 87)			Intervention group (*n* = 75)			Difference between groups	
	Pre	Post	*t*-test *p*-value	Pre	Post	*t*-test *p*-value	Pre	Post
	M SD	M SD		M SD	M SD		*t*-test *p*-value	*t*-test *p*-value
Time in school sport classes (min).	12.48 (19.88)	14.71 (19.91)	*t* = –1.78 *p* = 0.079	12.04 (18.77)	54.26 (14.74)	*t* = –16.08 *p* < 0.001**	*t* = 0.14 *p* = 0.885	*t* = –14.17 *p* < 0.001**
No. of days played or trained in activity.	0.47 (0.96)	0.71 (1.23)	*t* = –2.03 *p* = 0.046*	0.64 (1.07)	2.69 (0.94)	*t* = –13.23 *p* < 0.001**	*t* = –1.05 *p* = 0.293	*t* = –11.36 *p* < 0.001**
Total estimated time participating in organized sport (min).	12.59 (29.27)	22.87 (41.39)	*t* = –42.40 *p* = 0.019*	13.12 (26.56)	56.24 (42.85)	*t* = –8.42 *p* < 0.001**	*t* = –0.12 *p* = 0.904	*t* = –5.03 *p* < 0.001**
No. of days participated in moderate physical activity.	1.18 (0.91)	1.48 (1.18)	*t* = –2.18 *p* = 0.032*	091 (1.14)	2.77 (1.42)	*t* = –10.35 *p* < 0.001**	*t* = 1.72 *p* = 0.087	*t* = –6.32 *p* < 0.001**
Doing vigorous exercise.	25.94 (22.88)	24.11 (26.06)	*t* = 0.54 *p* = 0.593	22.24 (34.36)	45.87 (36.59)	*t* = –4.38 *p* < 0.001**	*t* = 0.82 *p* = 0.415	*t* = –4.47 *p* < 0.001**
Time in using a computer, watching TV, watching videos or using the internet as part of study or homework (hr).	17.22 (2.64)	16.64 (2.48)	*t* = 1.64 *p* = 0.105	16.53 (2.10)	11.95 (3.11)	*t* = 21.27 *p* < 0.001**	*t* = 1.80 *p* = 0.073	*t* = 10.67 *p* < 0.001**
Time in using a computer, watching TV, watching videos/DVD, using PlayStation, using the internet or playing electronic games for fun and recreation (hr).	14.46 (2.42)	14.61 (2.64)	*t* = –0.44 *p* = 0.660	14.43 (2.31)	5.03 (1.57)	*t* = 18.93 *p* < 0.001**	*t* = 0.88 *p* = 0.930	*t* = 27.53 *p* < 0.001**
Time in sleeping, relaxing, sitting and socializing with friends and family outside of school time during the daytime (hr)	12.16 (1.45)	12.68 (2.00)	*t* = –2.29 *p* = 0.024*	12.08 (2.78)	4.64 (2.40)	*t* = 18.07 *p* < 0.001**	*t* = 0.23 *p* = 0.813	*t* = 23.19 *p* < 0.001**

M: Mean; SD: Standard Deviation.

Pre: pre-intervention. Post: post-intervention.

*p*-value: *significant at < 0.05 level; ** Significant at < 0.001 level.

Concerning dietary habits, after implementing the intervention, the children reduced unhealthy dietary habits, such as the number of meals, large meals, snacks of sweets/chocolates, and glasses of sweet fizzy drinks. Additionally, they increased their healthy dietary habits, such as the number of pieces of fruit, servings of vegetables, and glasses of water. Furthermore, there was a change in physical activity with increased time (including a number of days) spent in physical activities, such as school sports classes, dance, swimming, walking, and organized sports; in the number of days spent participating in moderate physical activity; and in the number of hours doing vigorous exercise. Along with this, there was a decrease in inactive exercises, including time spent using a computer, watching TV, watching videos, using the Internet as part of study or homework, using PlayStation, using the Internet or playing electronic games for fun and recreation, sleeping, relaxing, and sitting, and socializing with friends and family outside of school time (Tables 4 and 5).

## Discussion

The aim of the current study was to assess the effect of a family-based lifestyle intervention on reducing body weight among Jordanian children with obesity aged 6 to 9 years old. This study showed a statistically significant change in the BMI percentile and median BMI percentile scores of children after they participated in an intervention for weight reduction. In addition, statistically significant changes in dietary habits and physical activity were observed after the intervention, in which the dietary habits were modified, and the level of physical activity was increased. The results are consistent with the previous evidence reporting the effectiveness of family-based lifestyle interventions in the management of childhood obesity (37, 38).

There was a change in the BMI percentile and median BMI percentile after the intervention among the intervention group. This means that the average BMI percentile and median BMI percentile scores in the intervention group reduced by a clinically significant amount, especially among the participants who are in an age group that is sometimes difficult to control or reduce their weight in such a short period (3-month). Also, those children were in a growing period; therefore, the cornerstone of this intervention was to modify dietary habits and physical activity during this growth period and minimize fat mass, which results in more active and healthy behaviors that minimize the negative results of obesity and overweight. Another study, however, has found no effects of implementing a family-based lifestyle intervention to reduce BMI percentile among children aged 4–8 years in Netherlands (39). The possible explanation for the different outcomes of the two studies could be related to age groups and nature of the intervention used in the studies.

The current study targeted promoting healthy food habits and physical activity in children. However, one research (32) has shown that the majority of children consumed fewer nutritious foods, such as fruits and vegetables, demonstrated a low to moderate physical activity level, and watched TV for more than an hour daily. Therefore, developing an intervention targeting the sedentary lifestyle of children is valid, to prevent childhood obesity and possible future disease (40). There is a need for interventions that target healthy lifestyles to overcome negative sedentary behaviors among children, parents, families, and schools. Previous study has found a positive significant relationship between parental obesity and their children’s obesity, and vice versa (9). One previous study showed that supportive parents were a significant positive predictor of children meeting sufficient levels of physical activity and healthy eating habits (41). An early study found that family-based interventions are strongly recommended to be effective, especially if this intervention targeted all family members (42).

Interventions need to have different behavioral techniques to keep participants on track and following the given instructions to achieve the targeted goal. The American Heart Association and the National Institute for Health and Care Excellence signify the importance of establishing behavioral strategies that are sensitive to children’s needs to promote their health. For example, there is a need to evaluate beliefs and tacit knowledge that is harbored by children and their parents toward developing sensitive programs that would modify their behavior-associated activities (43–46). A recent integrative review found that school-aged children have the ability to detail facts about food and health. They are also able to identify the main reasons influencing their decisions about eating and barriers to being healthy and having a good appearance (47). Therefore, it is necessary to include such information in the children’s education curriculum at this stage to influence their future decisions and overcome the issue of obesity.

Another important element in any successful intervention is physical activity, which was identified as one of the main responsible modifiable risk factors of obesity (48). Children should engage in moderate to intensive physical activities for at least 1 h every day (49). To accomplish this, decision-makers in schools should follow the school curriculum to ensure that children take the time allotted for physical activity as recommended by the WHO (49). Also, in homes, parents should encourage and motivate their children to exercise. Furthermore, there is a need to arrange a proper environment for practicing physical activity, whereas there is a lack of recreational areas, which blocks children and their families from exercising. Therefore, there is a need to designate more available free lands, playgrounds footways, and parks for children and their families to play and expend their energy (23–25). Current evidence has shifted to blaming the built environment instead of blaming people for their sedentary lifestyle. If the environment is not supportive, people cannot change their lifestyle (23).

The important aspects of the intervention’s success in the current study are the adherence and continuity of this intervention for 3 months and the involvement of parents in supporting their children. It is anticipated that family members will continue to support each other, so these good behaviors and practices will likely continue. It is recommended to include teachers in future research to combine family-based interventions and school-based interventions, which could maximize the effect. Healthcare professionals, including nurses and dieticians, can play a significant role. Nurses can be a support in developing tools, such as advocacy, leadership, and marketing skills, that will play an effective role in the prevention of childhood obesity and in implementing the best intervention for weight loss.

The current study had many strengths, including the use of a randomized design, a control group, and a large sample size, as well as implementing a family-based lifestyle intervention. Future studies should be conducted to follow-up with the children and to assess the improvements in their BMI percentile and median BMI percentile, dietary habits, and physical activities, according to age, gender, and parents’ educational level.

However, there are a few limitations that need consideration, including self-reported data related to diet and physical activity, which could be subjective. This intervention was educational and focused on providing information about dietary habits and physical activity with little focus on psychological methods; therefore, any future intervention should be implemented integrating behavioral methods. Further studies could be conducted to assess the well-being and adverse consequences of such an intervention that targets children.

## Conclusion

The prevalence of childhood obesity is rising. The study results suggest that family-based lifestyle interventions can have a statistically significant effect on BMI percentile and median BMI percentile scores, dietary habits, and physical activity among children with obesity who were aged 6 to 9 years old in the intervention group, but no effect on the BMI percentile among the children in the control group.

The results can help healthcare professionals, public health workers, and policymakers adopt such interventions for children with obesity in their community, especially through schools. Also, further research is needed to evaluate the effectiveness of this intervention in the short and long terms. In addition, future intervention strategies need to take into account other factors including the environment (e.g., food preparation, meal patterns, and use of the Internet and video games) and the role of schools in implementing such interventions.

## Implications

Decision-makers in schools should follow the school curriculum to ensure that children take the time allotted for physical activity as recommended by the WHO. It is recommended that a parental program should be developed related to healthy eating and physical activity using a family-focused approach. Nurses play a vital role in educating and counseling both children and their families concerning the significance of dietary habits and physical activity and emphasize the importance of continuous support and reinforcement of healthy lifestyle practices in children’s lives.

## Data Availability

The data are available, but they are confidential. As a result, it cannot be provided but on request.

## Author agreement

All authors have seen and approved the final version of the manuscript being submitted. They warrant that the article is the authors’ original work, has not received prior publication, and is not under consideration for publication elsewhere. All authors declare that all participants in this study gave their written informed consent to participate. All authors gave final approval and agree to be accountable for all aspects of the work, ensuring integrity and accuracy.
